# Clinical outcomes after mix-and-match implantation of monofocal and refractive multifocal intraocular lenses after surgery

**DOI:** 10.3389/fmed.2025.1579025

**Published:** 2026-01-22

**Authors:** Ying Xing, Fuqiang Li, Yaru Wang, Zengfei Ma, Zengjie Wang, Hui Zhang

**Affiliations:** 1 Shaanxi Provincial Rehabilitation Hospital, Shaanxi, China; 2 The Second Hospital of Jilin University, Changchun, China

**Keywords:** cataract surgery, partial monovision, refractive surgery, stereo vision function, visual quality

## Abstract

**Purpose:**

To analyze the visual acuity, visual quality, and stereoscopic function of patients with binocular cataracts following mixed refractive multifocal intraocular lens (MIOL) (Lentis Comfort LS-313 MF15; Oculentis GmbH, Germany) and monofocal IOL (Tecnis ZCB00; Johnson & Johnson Vision, USA) implantation.

**Setting:**

The study was conducted at the Eye Center of our hospital between January 2023 and December 2023.

**Design:**

This was a retrospective, case–control study.

**Methods:**

This retrospective study compared mixed implantation (LS-313 MF15 in non-dominant eye + ZCB00 in dominant eye, *n* = 30) versus bilateral monofocal implantation (ZCB00, *n* = 30). Visual acuity (uncorrected distance visual acuity (UDVA), uncorrected intermediate visual acuity (UIVA), uncorrected near visual acuity (UNVA)), defocus curves, stereopsis, and patient-reported outcomes were assessed at 3 months.

**Results:**

Sixty patients (120 eyes) were enrolled. Group A exhibited significantly better UIVA (*p* < 0.001) and UNVA (*p* < 0.001) than Group B. The defocus curve indicated more continuous visual acuity in Group A, which had a significantly better postoperative VF-14 (Visual Function Index-14) score (*p* < 0.001) and spectacle independence rate than Group B. Near-stereoscopic acuity measured using Titmus card showed clearer stereoscopic vision in Group A than in Group B, with a significant difference (*p* < 0.001).

**Conclusion:**

Hybrid monovision may be an effective approach for patients with binocular cataracts since the concept of partial monovision is well-suited for the desire of eyeglass independence.

## Introduction

The diversified development of IOL function has ushered cataract surgery into the era of refractive surgery (1). Historically, although monofocal IOLs provided good distance vision, acquiring intermediate and near vision still required corrective glasses post-surgery (2, 3). Over the past few decades, the clinical application of MIOLs, based on diffraction or refraction principles, has provided patients with cataracts with improved near and distance vision post-surgery (4–8); however, intermediate vision remains inadequate. Moreover, with the advent of the information age, people’s work and lives have become increasingly dependent on electronic devices, escalating the demand for intermediate vision.

The Lentis Comfort LS-313 MF15 (9, 10)is a rotary asymmetrical MIOL with a + 1.50 D attached to it, providing not only good intermediate and distance vision but also good near vision, showing a trend of continuous visual range. Although monofocal IOLs can mitigate postoperative adverse optical phenomena to a certain extent, they can cause visual fatigue, loss of stereoscopic vision, and inadequate near and intermediate vision, highlighting their limitations (11).

Therefore, in this study, we aimed to use mixed binocular implantation of a monofocal IOL, ZCB00, and regional refraction MIOL, LS-313 MF15. The combination of both methods, monovision and the implantation of an MIOL in one eye, leads to a modified monovision: a partial monovision (12). In this procedure, one eye receives a monofocal IOL and the other eye a multifocal system (13). To minimize the limitations of monofocal IOLs, harness their respective advantages, and improve postoperative visual acuity, visual quality, and patient satisfaction.

## Methods

### Study participants

This retrospective, case–control study included 60 (120 eyes) patients with cataracts, aged 34–75 years, who underwent phacoemulsification combined with IOL implantation at the Eye Center of our hospital between January 2023 and December 2023. Table 1 presents the baseline patient data before surgery. A posterior capsule light examination was performed 3 months postoperatively, revealing no significant events affecting visual acuity or refractive status due to posterior capsule opacity. This study was reviewed and approved by the Ethics Committee of The Second Hospital of Jilin University (Research and Approval No. 125 in 2024). All patients signed informed consent forms after explanation of the nature and possible consequences of the study, and the study was approved by the institutional review board. Additionally, this study adhered to the tenets of the Declaration of Helsinki.

**Table 1:** Demographic characteristics and preoperative indicators of patients.

Preoperative parameter	Mean ± SD range: (*X*–*X*)		*p* value
	Group A	Group B	
Patients (eyes)	30 (60)	30 (60)	–
Gender (male/female)	10/20	10/20	1.000
Age (years)	65.73 ± 9.82 (41 to 75)	61.33 ± 8.69 (34 to 75)	0.566
AL (mm)	23.25 ± 1.08	23.11 ± 0.98	0.177
ACD (mm)	2.96 ± 0.39	2.85 ± 0.34	0.294
CECC (cells/mm^2^)	2627.73 ± 374.49	2544.45 ± 416.58	0.341
IOP (mmHg)	16.32 ± 2.13	18.12 ± 2.78	0.092
UDVA (log MAR)	0.79 ± 0.54	0.73 ± 0.44	0.062
CDVA (log MAR)	0.69 ± 0.52	0.70 ± 0.45	0.241

Group A, mixed-implantation group; Group B, bilateral monofocal group; SD, standard deviation; AL, axial length; ACD, anterior cell depth; CECC, corneal endothelial cell count; IOP, intraocular pressure; UDVA, uncorrected distance visual acuity; CDVA, corrected distance visual acuity.

### Grouping, inclusion, and exclusion criteria

The study cohort was equally divided into two groups (30 patients/60 eyes per group). Group A (mixed-implantation group) received a monofocal IOL ZCB00 in the dominant eye first and MF15 in the non-dominant eye. Group B (bilateral monofocal group) received ZCB00 IOLs in both eyes. All patients underwent sequential bilateral cataract surgery, with the second eye procedure performed within 2 weeks of the first eye surgery in both groups. Postoperative evaluations commenced 3 months after the second eye surgery to ensure stabilization of refractive outcomes and complete neuroadaptation.

Ocular dominance was determined preoperatively using the +1.50 D lens fogging method combined with the hole-in-card test. Specifically, with both eyes open and fixating on a distant target, which was a single line of letters at 5 m, a +1.50 D spherical lens was placed before the non-tested eye to blur its vision. The patient was then asked to view the same distant target through a small aperture in the center of a card. The patient was instructed to indicate the position of the target seen. The card was then removed, and the examiner verified that the target indicated by the patient was the same one seen through the aperture. This process was repeated several times. The eye that was consistently used to align with and identify the target was designated as the dominant eye. Group A was comprised of patients who made an informed personal choice for this treatment strategy. Each patient in Group A demonstrated a strong personal motivation to reduce spectacle dependence and, after passing a rigorous preoperative suitability assessment, provided fully informed consent. This consent process explicitly covered the potential benefits and risks of the mixed-implantation approach. Patients understood that the goal was to achieve functional binocular vision through complementary focus, not perfect full-range vision in each eye individually.

Inclusion criteria were as follows: (1) patients diagnosed with cataracts before surgery, aged 30–75 years; (2) regular corneal astigmatism within 1.0 D; (3) kappa angle and alpha angle < 0.5; and (4) bright adaptive pupil > 2.0 mm, dark adaptive pupil < 6.0 mm. The exclusion criteria were as follows: (1) history of eye surgery, trauma, uveitis, retinopathy, glaucoma, high myopia, or severe dry eye; (2) irregular corneal astigmatism; (3) intraoperative complications; and (4) diabetes, immune disease, systemic disease, or inability to follow the interview schedule. (5) Patients who could not undergo reliable ocular dominance determination.

### Preoperative examination

All patients underwent a comprehensive ophthalmic examination before surgery, including corrected vision, corneal endothelial count, optical coherence tomography (OCT), slit-lamp assessment, IOL Master 700 measurements, and mydriatic fundoscopy.

### Surgical technique

All surgeries were performed by the same experienced surgeon (HZ). IOL power was chosen to target emmetropia (− 0.5 D). Levofloxacin eye drops (0.5%) were administered to both eyes of the patients 3 days before surgery, and the pupils were dilated with compound tropicamide eye drops 30 min before surgery. After surface anesthesia, a 3.0-mm incision was made at the 1–1.5-mm medial limbus of the operative eye at the 10–11 o’clock position. An additional incision was made with a 15° scalpel, the anterior chamber was injected with a viscoelastic agent, and the anterior capsule was subjected to continuous curvilinear capsulorhexis with a diameter of approximately 5–6 mm. Hydrodissection and delamination were performed, followed by phacoemulsification using a phacoemulsification device to remove the lens nucleus. Subsequently, the residual cortex was aspirated using an irrigation/aspiration handle. A viscoelastic agent was injected into the capsule, and the IOL was implanted. The residual viscoelastic agent was aspirated, followed by a watertight corneal closure. Dexamethasone ophthalmic ointment was then administered to the conjunctival sac, and a sterile dressing was applied to the operative eye. Anti-inflammatory therapy was subsequently recommended for postoperative care.

### Evaluation of postoperative visual quality

Binocular visual acuity (UDVA, UIVA, UNVA), binocular defocus curve, and VF-14 (14, 15) of the two groups were measured 3 months after surgery. A quality of vision (QoV) (16) questionnaire was administered, and stereoacuity was evaluated using the Titmus test and Distance Randot Stereotest. Technicians responsible for collecting key objective outcome measures using standard logarithmic visual acuity charts, as well as defocus curves obtained with an autorefractor, were unaware of the patients’ group assignments. All results were obtained either directly from the device or recorded by the examiner to ensure maximal neutrality in objective assessment. For the questionnaire, we used standardized self-assessment tools. Patients completed these independently in a separate setting or, when assistance was needed, with the help of a research assistant not involved in group allocation, who used neutral language to avoid assessor-induced influence.

### Visual acuity measurement

Three months post-surgery, UDVA was measured using a standard logarithmic visual acuity chart at a distance of 5 m. UNVA and UIVA were measured using a standard near-vision chart at 40- and 80-cm distances, respectively. All measurements were conducted under consistent lighting conditions, and visual acuity values were converted to logarithm of the minimum angle of resolution (log MAR) values for statistical analysis.

### Defocus curve analysis

The defocusing curve (17, 18) was drawn, and the degree of the spherical lens was adjusted using a comprehensive optometer. When the diopter was not corrected, the spherical lens degree was decreased from +2.0 D to −5.0 D, and the visual acuity of the operative eye was obtained at different diopters by decreasing the spherical lens degree by 0.5 D. Comparison of postoperative Spectacle independence rate and adverse optical interference.

### Questionnaire

The QoV questionnaire was used to investigate visual quality. The results identified glare, halo, starlight, and blurred vision as the four types of adverse optical interference phenomena. This instrument is monocularly assessed. In this study, we strictly adhered to its standard protocol: during evaluation, the patient’s non-tested eye was occluded, and they reported the frequency and severity of adverse optical phenomena (e.g., glare, starburst, halo) based on the visual experience of one eye only. Therefore, each patient (if both eyes were operated on) completed the QoV assessment twice, independently for each eye. For statistical analysis, we treated the “eye” as the unit and employed models using the signed-rank test to account for the correlation between the two eyes of the same patient. The VF-14 questionnaire (19) evaluated the postoperative visual function following cataract surgery. This instrument assesses binocular overall function. It inquires about the difficulty a patient experiences in performing 14 common daily activities under natural binocular viewing conditions, which are divided into five levels based on the degree of difficulty. The average value of each item was summed, and the total was multiplied by 25. The rate of postoperative spectacle independence rate was also recorded. Spectacle independence means that patients do not require the use of spectacles for any daily activity, including distance, intermediate, and near vision.

### Stereoscopic acuity examination

A Titmus stereoscopic card was used to check the patient’s near-distance stereoscopic acuity (20), which includes 3,000 “big flies” and 800–20 “circles.” The “big fly” qualitatively checks for stereo qualitatively, while the “circles” provide quantitative measurements. During the examination, patients were required to wear polarized light glasses and were positioned 40 cm from the examination book. Randot random point stereograms were used to measure long-range stereoacuity from a distance of 3 m (21).

### Statistical methods and calculations

Statistical analysis was performed using SPSS 25 (IBM, Armonk, NY, USA). The Kolmogorov–Smirnov test was used to test the normality of data distribution. The Student’s *t*-test for independent samples was used for normally distributed data, with results expressed as mean ± standard deviation. The Mann–Whitney *U* test was performed to evaluate differences between two independent samples for non-normally distributed data. For comparisons of paired or matched observations (e.g., the two eyes of the same patient), the Wilcoxon signed-rank test was employed. Fisher’s exact or the chi-squared (*χ*^2^) test was used to determine the ratios between the two groups. All tests were two-tailed, and statistical significance was set at *p* < 0.05.

## Results

### Patient characteristics

A total of 60 patients (120 eyes) were included in this study. The average age of patients was 65.73 ± 9.82 years in Group A and 61.33 ± 8.69 years in Group B. No statistically significant differences were observed in age, eye type, sex, corneal astigmatism, axial length, or intraocular pressure between the two groups (*p* > 0.05) (Table 1).

### Postoperative visual acuity statistics

No significant difference was observed in the UDVA between the two groups 3 months after surgery (*p* = 0.5). Group A exhibited significantly better binocular UIVA and UNVA than those in Group B (both *p* < 0.001; Tables 2, 3).

**Table 2:** Summary of postoperative visual acuity in group A in 3 months follow-up mean ± SD.

Parameter	ZCB00	MF15	BVA-Group A	*F*	*p* value			
					I	II	III	IIII
UDVA (logMAR)	0.047 ± 0.051	0.051 ± 0.055	0.040 ± 0.047	0.323	0.725	–	–	–
UIVA (logMAR)	0.296 ± 0.129	0.189 ± 0.146	0.135 ± 0.117	11.688	<0.001^*^	0.004^**^	0.122	<0.001^**^
UNVA (logMAR)	0.451 ± 0.104	0.349 ± 0.140	0.315 ± 0.134	9.225	<0.001^*^	0.002^**^	0.341	<0.001^**^

BVA-Group A, bilateral visual acuity of mixed-implantation group; UDVA, uncorrected distance visual acuity; UIVA, uncorrected intermediate visual acuity; UNVA, uncorrected near visual acuity; I, ZCB00 vs MF15 vs BVA-Group A; II, ZCB00 vs MF15; III, MF15 vs BVA-Group A; IV, ZCB00 vs BVA-Group A.

* Statistically significant (*P* < 0.05) by ANOVA.

** Statistically significant (*p* < 0.017) after Bonferroni correction.

**Table 3:** Comparison of postoperative postoperative binocular visual acuity and VF-14 score between Group A and Group B in 3 months follow-up mean ± SD.

Parameter	Group A	Group B	*p* value
UDVA (logMAR)	0.040 ± 0.047	0.049 ± 0.054	0.500
UIVA (logMAR)	0.135 ± 0.117	0.348 ± 0.085	<0.001^*^
UNVA (logMAR)	0.315 ± 0.134	0.476 ± 0.084	<0.001^*^
VF-14	92.02 ± 6.11	70.83 ± 6.16	<0.001^*^

Group A: Mixed-implantation group; Group B: Bilateral monofocal group; UDVA: uncorrected distance visual acuity; UIVA: uncorrected intermediate visual acuity; UNVA: uncorrected near visual acuity; VF-14: visual function index scale.

* Statistically significant (*p* < 0.05).

### Questionnaire

A comparison of the postoperative VF-14 scores showed higher scores in Group A than in Group B (*p* < 0.05). Evaluation of adverse optical interference—glare, starburst, halo, and blurred vision—revealed no significant differences between the two groups (*p* > 0.05) (Table 3). The QoV questionnaire specifically evaluated the incidence of glare, starbursts, halos, and blurred vision, with severity graded as: never, mild, moderate, and severe. A total of 60 patients (120 eyes) were included in this study, comprising 30 multifocal eyes and 90 monofocal eyes. Non-parametric tests were employed to analyze the ordinal symptom grade data. For the independent samples comparison between eyes with multifocal intraocular lenses (*n* = 30) and all eyes with monofocal lenses (*n* = 90), the Mann–Whitney *U* test revealed no statistically significant differences in the severity of any adverse visual phenomena between the two groups: glare (*Z* = −1.381, *p* = 0.167), starburst (*Z* = −1.488, *p* = 0.137), halo (*Z* = −1.707, *p* = 0.088), and blur (*Z* = −1.401, *p* = 0.161). Furthermore, a paired analysis of the two eyes within the same patients in Group A was conducted. The results of the Wilcoxon signed-rank test indicated no significant differences in symptom severity between the multifocal eyes and their contralateral monofocal eyes: glare (*Z* = −0.541, *p* = 0.589), starburst (*Z* = −0.992, *p* = 0.321), halo (*Z* = −1.231, *p* = 0.218), and blur (*Z* = −1.000, *p* = 0.317). (Tables 5, 6).

The postoperative spectacle independence rate was notably higher in Group A (93.3%) than that in Group B (23.3%). Only a small number of patients chose to occasionally use low-power reading glasses (approximately +1.00 D) during near vision tasks to optimize comfort, which represents an optional enhancement rather than a necessary correction, indicating that most patients in Group B continued to wear glasses for close and detailed tasks, as reported during the postoperative follow-up.

### Defocus curve

**Figure 1:**
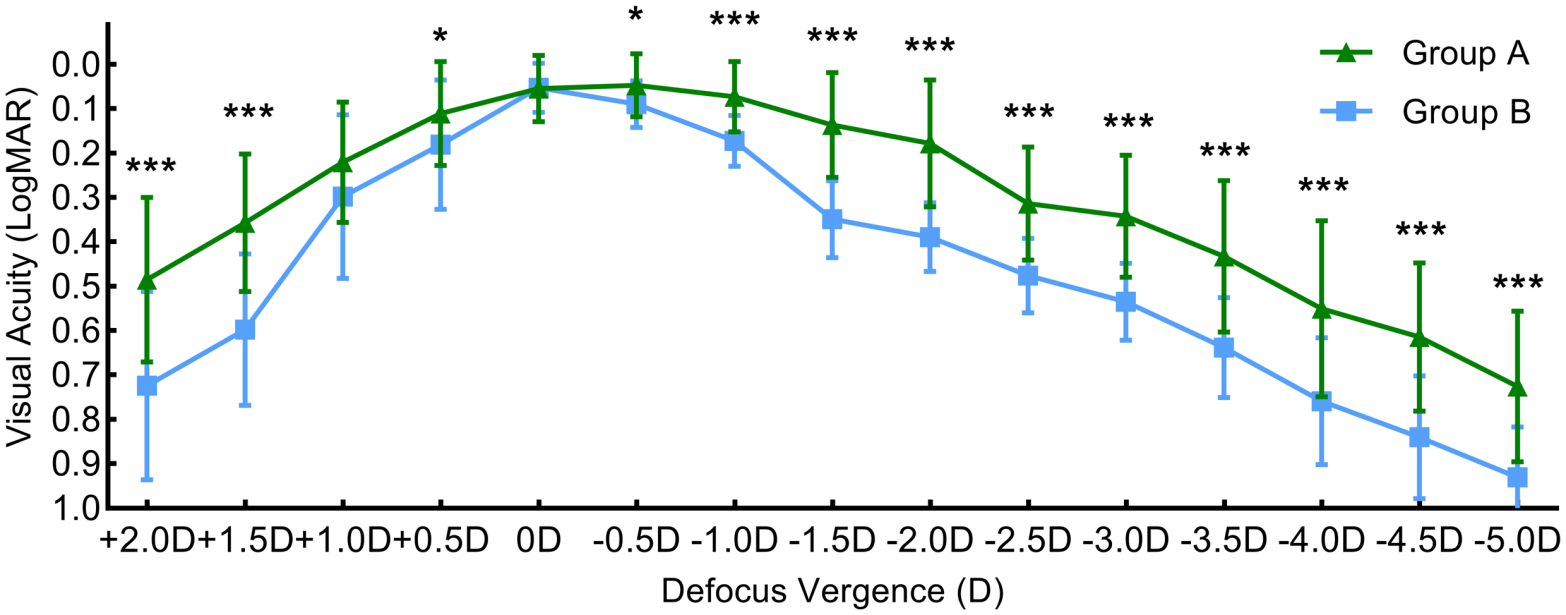
The Defocus Vergence (D) of both of eyes of group A and group B.

In Group A, the defocus curve of both eyes gradually increased from +2.0 D to +0.5 D, reaching a plateau within the range of 0 D to −1.0 D with the average visual acuity above 0.1 logMAR. Visual acuity gradually decreased from −1.0 D to −5.0 D, with acuity above 0.4 logMAR in the range of −1.5 D to + 2.5 D. Conversely, the defocus curve for group B was unimodal, with peak visual acuity at 0 D and an average visual acuity above 0.1 logMAR. The visual acuity of Group A was superior across the range of 0 D to −5.0 D compared to that of Group B (Figure 1).

### Stereopsis function

Near-stereoscopic acuity was assessed using a Titmus stereoscopic view card, while long-distance stereoscopic acuity was evaluated using a Randot random-point stereogram at 3 m. Results indicated that 93.33 and 76.67% patients in Group A achieved 60″ for long-distance stereoscopic acuity and near-stereoscopic acuity compared to 90 and 23.33% patients in Group B, respectively. Patients in both groups demonstrated good long-range stereovision without statistically significant differences. Group A exhibited significantly better near-distance stereovision than did Group B (*p* < 0.001; Table 4).

**Table 4:** Comparison of distance and near stereoscopic between Group A and Group B.

Parameter	Group A	Group B	*Z*	*P* value
Distance stereoscopic acuity	60 (60,60)	60 (60,60)	−0.494	0.622
Near stereoscopic acuity	32 (25,50)	100 (63,200)	−4.414	<0.001^*^

Group A: Mixed-implantation group; Group B: Bilateral monofocal group.

* Statistically significant (*P* < 0.05).

**Table 5:** Comparison of adverse visual phenomena between multifocal and monofocal IOLs in both group.

Parameter	MF15 (*n* = 30) (never/mild/moderate/severe)	ZCB00 in both Group (*n* = 90) (never/mild/moderate/severe)	*Z*	*P* value
Glare	25/4/1/0	83/5/2/0	−1.381	0.167
Starburst	24/3/1/2	81/6/2/1	−1.488	0.137
Halo	22/5/2/1	78/8/3/1	−1.707	0.088
Blur	26/3/1/0	85/4/1/0	−1.401	0.161

Analysis was performed using Mann–Whitney *U* test.

**Table 6:** Paired comparison of adverse visual phenomena between multifocal and monofocal IOLs in Group A.

Parameter	MF15 (*n* = 30) (never/mild/moderate/severe)	Contralateral ZCB00 in Group A (*n* = 30) (never/mild/moderate/severe)	*Z*	*P* value
Glare	25/4/1/0	27/2/1/0	−0.541	0.589
Starburst	24/3/1/2	26/3/1/0	−0.992	0.321
Halo	22/5/2/1	25/4/1/0	−1.231	0.218
Blur	26/3/1/0	28/2/0/0	−1.000	0.317

Analysis was performed using Wilcoxon signed-rank test.

## Discussion

With the rapid advancement and evolution of IOL materials and functionalities, refractive cataract surgery has progressively become mainstream in cataract treatment. Unlike traditional refractive procedures, refractive cataract surgery prioritizes comprehensive visual outcomes, including postoperative far, intermediate, and near vision, as well as overall visual quality (22, 23). Different optical design principles suggest that MIOLs can be classified as refractive, diffractive, and refractive–diffractive. Additionally, MIOLs provide superior near vision compared to monofocal IOLs and are increasingly used in clinical practice. According to the literature (24), most patients undergoing cataract surgery typically expect improved visual quality at the same time as postoperative lens removal. Therefore, in recent years, the design technology and application rate of MIOLs have rapidly increased (25).

In contrast, traditional monofocal IOLs have less optical interference after surgery. However, Singh et al. showed that they can only provide long- or short-distance monofocal vision, which does not meet diverse patient needs. Therefore, in recent years, MIOLs have increasingly been used. However, Bartol-puyal et al. (26) showed that their visual effect is affected by pupil size, and their specialized optical design may induce undesirable optical interference symptoms, such as glare, halos, and reduced contrast sensitivity. Furthermore, MIOLs are unsuitable for patients with eye diseases such as glaucoma, diabetic retinopathy, and age-related macular degeneration, which limits their clinical use. Therefore, MIOLs cannot completely replace monofocal IOLs.

The Lentis Comfort LS-313 MF15 is an aspherical regional refraction MIOL. Its features include: (1) hydrophilic acrylic material with surface hydrophobic treatment, which can effectively prevent the occurrence of later cataracts and postoperative endophthalmitis risks; (2) the design principle of a bifocal lens, where the top is the far area of view, bottom is the near area of view, and near additional diopter is +1.50 D. This low additional design causes the focal segments of the two focal points to coincide, thereby extending the focal depth and enhancing middle-distance visual acuity, resulting in a continuous visual range for far and intermediate distances. Additionally, two wedge transition areas are found in the middle, which facilitate the smooth transition between far and near vision. The light energy loss rate is only 5%, which improves contrast sensitivity and reduces adverse optical interference phenomena, such as glare and halo; (3) the rotary asymmetrical design strongly tolerates high-order aberrations, kappa angles, and alpha angles; (4) the wide optical focal range of −10.0 D to +35.0 D offers patients with highly myopic cataract an option for functional IOL; and (5) the regional refraction design principle without diffraction rings ensures it does not affect the posterior section of the surgery, fundus observation, or the laser treatments for the fundus and can be applied to patients with early diabetes.

The ZCB00 IOL is a monofocal IOL on the TECNIS platform with the following characteristics: (1) a one-piece, UV-absorbing posterior chamber made of hydrophobic acrylic material (27); (2) high Abbe number material to reduce chromatic aberration; (3) a − 0.27 μm aspherical negative spherical aberration design on the front surface, offsetting the average human cornea’s + 0.27-μm spherical aberration and achieving zero spherical aberration correction for the entire eye; (4) a 360° square edge design on the rear surface to reduce the formation of post-onset cataract; (5) diamond cold lathe processing technology to avoid flash formation; and (6) healthy blue light for improving dark vision and ensuring healthy biological rhythms in the human body.

Our findings showed that patients in both groups achieved good corrected distance visual acuity after surgery, with no statistically significant difference. Group A showed significantly better uncorrected intermediate and near vision than Group B did (*p* < 0.001). In addition, Group A was significantly more spectacle-independent without increased perception of optical side effects, such as halos and glare, compared to Group B. The superior intermediate and near vision provided by LS-313 MF15 in Group A met patients’ needs for spectacle independence.

The success of mixed implantation in improving UNVA and UIVA while maintaining good distance vision aligns with previous studies by Iida et al. on hybrid IOL strategies (13). The LS-313 MF15’s low-add design (+1.5 D) likely contributed to enhanced intermediate and near vision while minimizing dysphotopsias, a known trade-off with higher-add MIOLs (28). The high spectacle independence rate in Group A supports the clinical utility of this approach, particularly for patients with active near-vision demands. These results demonstrated comparable spectacle independence rates to those reported by Wendelstein et al. with EDOF–monofocal mix-and-match implantation (29).

The absence of significant differences in halos and glare between groups suggests that the LS-313 MF15’s optical profile is well-tolerated when paired with a monofocal IOL. This contrasts with studies using higher-add MIOLs, which often report increased photic phenomena. The preservation of contrast sensitivity—implied by comparable distance vision and lack of subjective visual disturbances—further supports the safety of this mixed implantation strategy.

Examining the near and far stereovision function revealed that patients in both groups achieved good distant stereovision with no statistically significant difference. Additionally, Group A demonstrated significantly better near stereovision than Group B (*p* < 0.001). The combination of ZCB00 in the dominant eye and a low-add MIOL, LS-313 MF15 (+1.5 D) in the non-dominant eye may allow the visual system to integrate two slightly different focal points, enhancing depth perception at near distances (30). Hibbard et al. (31) found that the integration of two slightly different focal points in the visual system is a critical mechanism for enhancing depth perception, particularly at near distances. This process is facilitated by binocular vision, which leverages the disparities between the images projected onto each retina to provide precise and accurate estimates of depth. The brain may selectively suppress blur from the multifocal component at near, while still utilizing binocular cues for stereopsis. Therefore, when evaluating visual performance, one must consider both the optical design characteristics and the plasticity of the nervous system.

This study employed an innovative method of implanting a monofocal IOL in the dominant eye and an MIOL in the non-dominant eye for patients with cataracts in both eyes. This approach aimed to enable the brain to inhibit the high-order aberrations and adverse visual phenomena generated by the IOLs when patients look into the distance, resulting in better visual effects. Using a monofocal IOL with blurred vision weakens the dominant eye’s function, allowing the MIOL near-sight feature to provide intermediate and near vision, thereby improving the postoperative spectacle independence rate. This synergistic approach combines the advantages of both IOL types while minimizing their potential limitations. The rationale behind this approach lies in the neuroadaptive capabilities of the visual system, which can integrate and prioritize visual inputs from both eyes to achieve binocular summation. By strategically positioning the monofocal IOL in the dominant eye, the brain can rely on its superior distance vision while utilizing the MIOL in the non-dominant eye for tasks requiring intermediate and near focus (32). This method not only enhances spectacle independence but also reduces the need for neural adaptation, which is often a challenge with MIOLs (33). Furthermore, the use of a defocused monofocal IOL in the dominant eye diminishes its functional role in near vision, thereby minimizing competition between the two eyes and optimizing the visual experience across all distances (34).

The defocus curve can be used to simulate vision at different distances and evaluate the IOL’s adjustment range. In this study, the defocus curve for Group A increased gradually from +2.0 D to +0.5 D, with an average visual acuity above 0.1 log MAR in the range of 0 D to- 1.0 D and decreased gradually in the range of −1.0 D to −5.0 D, maintaining visual acuity above 0.4 log MAR in the range of −1.5 D to −2.5 D. This indicates that the LS-313 MF15 and ZCB00 IOLs can synergistically achieve good binocular far, intermediate, and near vision. In contrast, the curve was unimodal for Group B, with the best visual acuity at 0 D and an average visual acuity above 0.1 log MAR. Compared to Group B, the binocular defocus curve of Group A showed a smoother trend in the range of 0 D to −5 D, providing more continuous and better overall visual acuity, which was attributed to the design principle of LS-313 MF15.

The QoV assessment scale is a questionnaire that evaluates the visual quality and quality of life for patients (35) and subjectively shows patients’ satisfaction. Our findings showed that the incidence of adverse optical phenomena in the multifocal eye was not statistically different from that in the monofocal eye. In the rigorously controlled paired comparison of Group A, no statistically significant differences were found for adverse visual phenomena. Therefore, multifocal lenses can still be considered to provide acceptable visual quality within a mix-and-match strategy. This is despite previous reports of higher incidences of adverse optical disturbances, such as halos or glare, experienced with MIOLs than with monofocal IOLs (36). The low-add MIOL (LS-313 MF15) in mixed implantation may mitigate dysphotopsias typically associated with traditional MIOLs. Patient adaptation or neural suppression mechanisms may play a role in reducing perceived visual disturbances over time (37). The combination may allow the visual system to prioritize the clearer image from the monofocal eye for distance vision while still benefiting from near vision support. This binocular summation effect could help suppress optical side effects, as suggested by Kim et al. (38).

The postoperative VF-14 scores demonstrated a statistically significant difference between the two groups (*p* < 0.001). Group A achieved a mean score of 92.02 ± 6.11, while Group B scored 70.83 ± 6.16. The findings demonstrate moderate visual impairment during specific tasks (e.g., reading small print), increased reliance on spectacle correction, and potential limitations in low-light conditions, which are consistent with the results reported by Beltraminelli et al. (39). The VF-14 questionnaire, a validated instrument for assessing visual function and quality of life in cataract surgery patients, considers scores above 90 as a robust indicator of surgical success. This threshold reflects both satisfactory visual rehabilitation and high patient satisfaction with vision-related activities. The superior VF-14 outcomes in Group A suggest that the combined implantation approach may offer significant advantages in restoring visual function and enhancing overall quality of life.

In conclusion, this study investigated the visual acuity, visual quality, and proximal and distal stereoscopic vision in patients with binocular cataracts 3 months after surgery involving refractive MIOL LS-313 MF15 and monofocal IOL ZCB00. We found that this hybrid implantation method fully utilized the potential and advantages of the different lenses, significantly reduced adverse optical phenomena, improved the postoperative spectacle independence rate, and maintained far and near stereoscopic acuity. Thus, this approach provides a new personalized treatment plan for patients with cataracts, who previously received monofocal IOL implantation in one eye and sought lens removal during cataract surgery in the second eye.

### Value statement

#### What was known

Regional refraction is the main optical design principle of the LS-313 MF15, whereas diffraction is the main optical design principle of the ZCB00. The main limitations of multi-focus IOLs reported in the past are reduced contrast sensitivity, flash vision symptoms, and inability to provide satisfactory vision at intermediate distances, while monofocal IOLs are unable to satisfy both long-distance vision and short-distance vision (40).

The Lentis Comfort LS-313 MF15 is a presbyopia correction IOL with a new design concept designed to meet the needs of intermediate vision while providing patients with clear vision quality.

#### What this study adds

Hybrid monovision has been reported in the past, but no clinical observation of LS-313 MF15 and ZCB00 mixed implantation has been reported. This study introduces a novel mix-and-match implantation strategy, combining a refractive multifocal IOL in the non-dominant eye with a monofocal IOL in the dominant eye to create a partial monovision system—a significant departure from conventional bilateral same-type IOL approaches. It further innovates by comprehensively evaluating stereoscopic function using the Titmus test, an outcome neglected in prior studies, and employs patient-centered metrics, such as the VF-14 questionnaire, to better capture real-world visual quality and spectacle independence. By directly comparing this hybrid model against standard bilateral monofocal implantation, the research provides clinically relevant insights tailored to patients seeking functional vision across distances without glasses.

#### Study limitation

While our sample size met statistical thresholds for primary outcomes, larger multicenter studies are needed to validate subgroup analyses. We acknowledge the possibility of residual confounding due to the non-randomized design and recommend that future prospective randomized studies be conducted for further validation. Some subtle baseline differences may represent residual confounding factors. We have recommended that future prospective studies adopt stricter matching or randomization procedures to better control for these variables. The 3-month observation period, while sufficient for assessing initial refractive stability and neuroadaptation, may not capture long-term outcomes such as late-onset posterior capsule opacification or delayed visual disturbances. A longer follow-up would be valuable to confirm the durability of visual outcomes, particularly in patients with mixed IOL implantation. Nonetheless, our study is limited by the lack of a direct comparison to a bilateral trifocal or EDOF (Extended Depth of Field) IOL implantation. It can be compared with previous studies. Consistent with previous outcomes from bilateral EDOF IOL and trifocal IOL implantation, our results similarly demonstrated favorable UDVA and UNVA. Since most of the patients are elderly and the post-operative follow-up rate is low, the sample size is limited for a period of time. Therefore, we suggest the accumulation of more samples by extending the study time in future studies.

## Data Availability

The raw data supporting the conclusions of this article will be made available by the authors, without undue reservation.

## References

[1] Narang R Agarwal A. Refractive cataract surgery. Curr Opin Ophthalmol. (2024) 35:23–7. doi: 10.1097/ICU.0000000000001005, 37962881

[2] Ohashi T Fujiya A Yoshida M Kojima T. Comparison of stereoacuity across distances in bilateral intraocular lens implantation: Monofocal versus diffractive multifocal lenses. Clin Ophthalmol. (2025) 19:2325–32. doi: 10.2147/OPTH.S527617, 40693282 PMC12277180

[3] Min S Won YK Kwon S-H Lim DH. Cost-benefit analysis of a trifocal intraocular lens versus a monofocal intraocular lens in South Korea. Sci Rep. (2025) 15:17330. doi: 10.1038/s41598-025-00712-0, 40389442 PMC12089271

[4] Nemet A Kanclerz P Tuuminen R. Should multifocal intraocular lenses become a standard in phacoemulsification cataract surgery? J Clin Med. (2023) 12:1983. doi: 10.3390/jcm12051983, 36902768 PMC10004625

[5] Labiris G Panagis C Ntonti P Konstantinidis A Bakirtzis M. Mix-and-match vs bilateral trifocal and bilateral EDOF intraocular lens implantation: the spline curve battle. J Cataract Refract Surg. (2024) 50:167–73. doi: 10.1097/j.jcrs.0000000000001336, 37847127

[6] Tavassoli S Ziaei H Yadegarfar ME Gokul A Kernohan A Evans JR . Trifocal versus extended depth of focus (EDOF) intraocular lenses after cataract extraction. Cochrane Database Syst Rev. (2024) 7:CD014891. doi: 10.1002/14651858.CD014891.pub2, 38984608 PMC11234495

[7] van den Berg AB van den Berg RM Rocha KM Chamon W Waring GO. Reading performance following contralateral implantation of an extended depth of focus (EDOF) IOL and a hybrid EDOF multifocal IOL. J Refract Surg. (2024) 40:e778–82. doi: 10.3928/1081597X-20240909-01, 39530986

[8] de Gracia P Dorronsoro C Marcos S. Multiple zone multifocal phase designs. Opt Lett. (2013) 38:3526–9. doi: 10.1364/OL.38.003526, 24104805

[9] Miyoshi M Tanabe H Shojo T Yamauchi T Takase K Tabuchi H. Comparison of visual performance between monofocal and rotationally asymmetric refractive intraocular lenses. PLoS One. (2025) 20:e0323625. doi: 10.1371/journal.pone.0323625, 40373074 PMC12080830

[10] Álvarez-García MT Rivera-Ruiz E Alió JL Piñero DP. Long-term prevalence of opacification of a hydrophylic acrylic rotationally asymmetric refractive multifocal intraocular lens. J Refract Surg. (2024) 40:e98–e107. doi: 10.3928/1081597X-20240115-01, 38346118

[11] Cho J-Y Won YK Park J Nam JH Hong J-Y Min S . Visual outcomes and optical quality of accommodative, multifocal, extended depth-of-focus, and monofocal intraocular lenses in presbyopia-correcting cataract surgery: a systematic review and bayesian network meta-analysis. JAMA Ophthalmol. (2022) 140:1045–53. doi: 10.1001/jamaophthalmol.2022.3667, 36136323 PMC9501783

[12] Knecht VA Colosi HA Hassenstein A. Partial monovision achieved by unilateral implantation of a multifocal add-on lens with bilateral pseudophakia: evaluation and results. Graefes Arch Clin Exp Ophthalmol. (2022) 260:2753–62. doi: 10.1007/s00417-022-05584-y, 35175409 PMC9325843

[13] Iida Y Shimizu K Ito M. Pseudophakic monovision using monofocal and multifocal intraocular lenses: hybrid monovision. J Cataract Refract Surg. (2011) 37:2001–5. doi: 10.1016/j.jcrs.2011.05.032, 22018364

[14] Chiang PP-C Fenwick E Marella M Finger R Lamoureux E. Validation and reliability of the VF-14 questionnaire in a german population. Invest Ophthalmol Vis Sci. (2011) 52:8919–26. doi: 10.1167/iovs.11-7702, 22025576

[15] Wan Y Zhao L Huang C Xu Y Sun M Yang Y . Validation and comparison of the national eye institute visual functioning questionnaire-25 (NEI VFQ-25) and the visual function index-14 (VF-14) in patients with cataracts: a multicentre study. Acta Ophthalmol. (2021) 99:e480–8. doi: 10.1111/aos.14606, 32940410 PMC8359188

[16] Rein AP Lundström M Dickman MM Rosen M Finkelman Y Semionov A . Assessing quality of vision in cataract surgery: randomized trial of digital vs paper-based questionnaires. J Cataract Refract Surg. (2025) 51:557–62. doi: 10.1097/j.jcrs.0000000000001642, 39999203

[17] Łabuz G Yan W Khoramnia R Auffarth GU. Comparing optical quality and simulated defocus curves: head-to-head analysis of hydrophilic and hydrophobic trifocal intraocular lenses. J Cataract Refract Surg. (2025) 51:161–6. doi: 10.1097/j.jcrs.0000000000001577, 39485924

[18] De Gracia P. Fourier tools for the evaluation of refractive multifocal designs. Sci Rep. (2023) 13:22585. doi: 10.1038/s41598-023-50172-7, 38114735 PMC10730910

[19] Wan Y Zhao L Huang C Xu Y Sun M Yang Y . Validation and comparison of the national eye institute visual functioning questionnaire-25 (NEI VFQ-25) and the visual function index-14 (VF-14) in patients with cataracts: a multicentre study. Acta Ophthalmol. (2021) 99:e480–8. doi: 10.1111/aos.14606, 32940410 PMC8359188

[20] Furr BA Musch DC Andrews CA Schumann R. Testability study of the titmus V3 test in pre-school children. Optom Vis Sci. (2018) 95:588–93. doi: 10.1097/OPX.0000000000001240, 29957735

[21] Nabie R Andalib D Khojasteh H Aslanzadeh SA. Comparison of the effect of different types of experimental anisometropia on stereopsis measured with titmus, randot and TNO stereotests. J Ophthalmic Vis Res. (2019) 14:48–51. doi: 10.4103/jovr.jovr_189_17, 30820287 PMC6388517

[22] Lapp T Wacker K Heinz C Maier P Eberwein P Reinhard T. Cataract surgery-indications, techniques, and intraocular lens selection. Dtsch Arztebl Int. (2023) 120:377–86. doi: 10.3238/arztebl.m2023.0028, 36794457 PMC10413970

[23] Ferguson TJ Randleman JB. Cataract surgery following refractive surgery: principles to achieve optical success and patient satisfaction. Surv Ophthalmol. (2024) 69:140–59. doi: 10.1016/j.survophthal.2023.08.002, 37640272

[24] Yeu E Cuozzo S. Matching the patient to the intraocular lens: preoperative considerations to optimize surgical outcomes. Ophthalmology. (2021) 128:e132–41. doi: 10.1016/j.ophtha.2020.08.025, 32882308

[25] Shen W Zhuo B Zhang L Shen J Ma D Yang J. Effect of astigmatism on visual outcomes after multifocal intraocular lens implantation: a systematic review and meta-analysis. Front Med (Lausanne). (2023) 10:1214714. doi: 10.3389/fmed.2023.1214714, 38089878 PMC10713711

[26] Bartol-Puyal F d A Talavero P Giménez G Altemir I Larrosa JM Polo V . Reading and quality of life differences between tecnis ZCB00 monofocal and tecnis ZMB00 multifocal intraocular lenses. Eur J Ophthalmol. (2017) 27:443–53. doi: 10.5301/ejo.5000925, 28106237

[27] Singh G Sidhharthan KS Reddy JK Sundaram V Thulasidas M. Comparison of visual outcomes in patients implanted with tecnis eyhance ICB00 and 1-piece ZCB00 monofocal intraocular lenses. Indian J Ophthalmol. (2024) 72:181–4. doi: 10.4103/IJO.IJO_681_23, 38273683 PMC10941920

[28] Alió JL Plaza-Puche AB Piñero DP Javaloy J Ayala MJ. Comparative analysis of the clinical outcomes with 2 multifocal intraocular lens models with rotational asymmetry. J Cataract Refract Surg. (2011) 37:1605–14. doi: 10.1016/j.jcrs.2011.03.054, 21855760

[29] Wendelstein JA Casazza M Reifeltshammer S Riaz K Pantanelli S Mariacher S . Unilateral intraindividual comparison and bilateral performance of a monofocal spherical and diffractive extended depth of field intraocular lens mix-and-match approach. Clin Experiment Ophthalmol. (2024) 52:31–41. doi: 10.1111/ceo.14315, 38050340

[30] Stewart EEM Valsecchi M Schütz AC. A review of interactions between peripheral and foveal vision. J Vis. (2020) 20:2. doi: 10.1167/jov.20.12.2, 33141171 PMC7645222

[31] Hibbard PB Haines AE Hornsey RL. Magnitude, precision, and realism of depth perception in stereoscopic vision. Cogn Res Princ Implic. (2017) 2:25. doi: 10.1186/s41235-017-0062-7, 28603771 PMC5442194

[32] Park ES Ahn H Han SU Jun I Seo KY Kim EK . Visual outcomes, spectacle independence, and patient satisfaction of pseudophakic mini-monovision using a new monofocal intraocular lens. Sci Rep. (2022) 12:21716. doi: 10.1038/s41598-022-26315-7, 36522397 PMC9755282

[33] Xiong T Chen H Fan W. Comparison of bilateral implantation of an extended depth of focus lenses and a blend approach of extended depth of focus lenses and bifocal lenses in cataract patients. BMC Ophthalmol. (2023) 23:476. doi: 10.1186/s12886-023-03228-1, 37990306 PMC10664382

[34] Tomagova N Elahi S Vandekerckhove K. Clinical outcomes of a new non-diffractive extended depth-of-focus intraocular lens targeted for mini-monovision. Clin Ophthalmol. (2023) 17:981–90. doi: 10.2147/OPTH.S405267, 37007049 PMC10053890

[35] Bellucci C Mora P Tedesco SA Gandolfi S Bellucci R. Comparison of objective and subjective visual outcomes between pentafocal and trifocal diffractive intraocular lenses. J Refract Surg. (2024) 40:e604–13. doi: 10.3928/1081597X-20240715-04, 39254241

[36] Moshirfar M Stapley SR Corbin WM Bundogji N Conley M Darquea IM . Comparative visual outcome analysis of a diffractive multifocal intraocular Lens and a new diffractive multifocal Lens with extended depth of focus. J Clin Med. (2022) 11:7374. doi: 10.3390/jcm11247374, 36555990 PMC9781237

[37] Zhang L Lin D Wang Y Chen W Xiao W Xiang Y . Comparison of visual neuroadaptations after multifocal and monofocal intraocular lens implantation. Front Neurosci. (2021) 15:648863. doi: 10.3389/fnins.2021.648863, 34194292 PMC8236945

[38] Kim B Son H-S Khoramnia R Auffarth GU Choi CY. Comparison of clinical outcomes between different combinations of hybrid multifocal, extended-depth-of-focus and enhanced monofocal intraocular lenses. Br J Ophthalmol. (2025) 109:565–71. doi: 10.1136/bjo-2024-325181, 39658143

[39] Beltraminelli T Rizzato A Toniolo K Galli A Menghini M. Comparison of visual performances of enhanced monofocal versus standard monofocal IOLs in a mini-monovision approach. BMC Ophthalmol. (2023) 23:170. doi: 10.1186/s12886-023-02920-6, 37085852 PMC10120133

[40] Fernández J Rocha-De-Lossada C Rodríguez-Vallejo M. Recommendations for writing clinical research manuscripts: from Monofocal to multifocal intraocular lenses. Int J Environ Res Public Health. (2022) 19:17036. doi: 10.3390/ijerph192417036, 36554917 PMC9778824

